# Gender-specific associations between neutrophil levels and refracture risks: a retrospective cohort study

**DOI:** 10.3389/fendo.2025.1625852

**Published:** 2026-01-13

**Authors:** Shuai Yuan, Xiao-jie Zhou, Hao-tian Jiao, Ya-qin Gong, Jian Jin, Yan Cao, Ke Lu, Chong Li

**Affiliations:** 1 Department of Orthopedics, Affiliated Kunshan Hospital of Jiangsu University, Suzhou, Jiangsu, China; 2 Kunshan Biomedical Big Data Innovation Application Laboratory , Suzhou, Jiangsu, China; 3 Information Department, Affiliated Kunshan Hospital of Jiangsu University, Suzhou, Jiangsu, China; 4 Kunshan Municipal Health and Family Planning Information Center, Suzhou, Jiangsu, China; 5 Department of Nuclear Medicine, Nanjing First Hospital, Nanjing Medical University, Nanjing, Jiangsu, China

**Keywords:** biomarkers, bone health, neutrophil levels, osteoporotic fractures, recurrent fractures, retrospective cohort study

## Abstract

**Background:**

Osteoporotic fractures (OPFs) significantly impact global health, predominantly affecting individuals over 50 years old. Furthermore, it has high incidence and refracture rates. Currently, the association between neutrophil (NEU) levels and the risk of recurrent fractures is still undetermined. Therefore, this study investigated the association between NEU levels at hospital admission and recurrent fracture risk in OPF patients.

**Methods:**

This retrospective cohort study analyzed 2,474 OPF patients who underwent surgical intervention at the Affiliated Kunshan Hospital of Jiangsu University between 2018 and 2023. The data were acquired from the hospital’s Osteoporotic Fracture Registration System. Patients’ NEUs were measured at admission, and they were followed up for recurrent fractures. Statistical assessments were carried out *via* Cox proportional hazards regression models to elucidate 5-year refracture risk. The nonlinear relationships were determined by smooth curve fitting and threshold analyses.

**Results:**

The results showed a significant sigmoidal (non-linear) relationship between the risk of recurrent fractures and NEU counts in male patients. Furthermore, NEU levels ranged between 4.4 - 8.5 ×10^9^/L indicated a substantially reduced risk of refractures (95% CI = 0.37 - 0.86; HR = 0.57; *P*-value = 0.008). Moreover, there were no substantial associations between NEU levels and refracture rates in the female cohort across all models.

**Conclusions:**

This study indicated a significant sigmoidal correlation between NEU levels and refracture risk in male OPF patients, ranging from 4.4 to 8.5 ×10^9^/L. Further research is required to elucidate these mechanisms and assess the clinical application of NEU levels as a biomarker for refracture risk in males.

## 1 Introduction

Osteoporotic fractures (OPFs) occur in bones that have become fragile due to osteoporosis, which increases bone’s susceptibility to breakage from low-energy impacts, such as minor falls (1). It has been estimated that there is an OPF incidence every 3 seconds worldwide, and its annual incidence is approximately 8.9 to 9 million (2). Furthermore, OPFs predominantly affect individuals over the age of 50, specifically postmenopausal women and elderly men. Moreover, they are also frequently observed in patients on long-term glucocorticoid therapy (3). OPFs have been observed to result in chronic pain and functional impairment, with severe cases requiring long-term care (4). The literature suggests that the mortality rate within one year of a hip fracture is about 20 - 24% (5). OPFs are a significant burden on the healthcare system and a major public health concern as they account for 50% of all fracture-related hospital admissions (6). Economically, the direct and indirect medical costs for OPFs are substantial, with global expenditures reaching at least $25 billion by 2025 (7). Therefore, it is crucial to enhance the prevention, early diagnosis, and treatment of osteoporosis to mitigate its extensive impacts.

Neutrophils (NEU) are pivotal leukocytes within the innate immune system, which eradicate invasive pathogens and participate in acute inflammatory responses (8). Furthermore, they influence bone health and pathology by releasing inflammatory mediators and enzymes (9). It has been observed that during the bone healing process, NEUs are among the first cells to arrive at the injury site to clear debris and infection for subsequent healing (10). This process involves the modulation of the inflammatory response and the recruitment of other immune cells, which collectively influence the healing trajectory. However, in bone-destructive diseases, such as rheumatoid arthritis, NEUs have also been found to exacerbate bone damage by promoting inflammation (11). Therefore, NEUs not only protect the body from infection but also regulate inflammatory responses and participate in bone reconstruction during bone fractures and other bone-related diseases. Overall, NEUs can serve as significant indicators of fracture prognosis and the risk of recurrent fractures.

Most previous literature was primarily focused on the association of the NEU-to-lymphocyte ratio (NLR) with fracture risk and neglected the continuous variation in NEU levels (12). Although NLR can reflect changes in inflammatory levels, its dependence on two independent indicators limits its effectiveness in directly assessing the link between fracture risk and NEU levels (13). In addition, the link between recurrent fracture risk and NEU levels in OPF patients remains unclear. Therefore, this retrospective cohort study aimed to elucidate the association between NEU levels and the risk of recurrent fractures in OPF patients during a long-term follow-up period.

## 2 Materials and methods

### 2.1 Data sources

Patient data was extracted from the Osteoporotic Fracture Registration System (OPFRS) established at the Affiliated Kunshan Hospital of Jiangsu University (AKHJU) in 2018 (14). The OPFRS prospectively records the clinical and demographic profiles of OPF patients, treatments administered, and outcomes, specifically those hospitalized for surgical interventions. This registry enables comprehensive data sharing and analysis across healthcare systems by integrating with the Regional Health Registration Platform of Kunshan City and the Population Death Registration System of Jiangsu Province.

This study only included patients aged ≥ 50 years who were treated at AKHJU for their first OPF between August 1, 2018, and August 30, 2023. The included patients had fractures of the proximal humerus, wrist, hip, and vertebrae, which are considered major sites for OPFs. The OPF diagnosis was based on the 10^th^ revision of the International Statistical Classification of Diseases and Related Health Problems (ICD-10; codes starting with S42, S32, S52, S22, or S72) (15). Furthermore, the diagnosis also followed the 2018 Chinese recommendations for the diagnosis and treatment of OPFs, emphasizing that low-energy or non-traumatic fractures caused by forces would not typically result in fractures (*e.g.*, falls from standing height or lower). Therefore, high-energy trauma-induced fractures were excluded from this study.

The OPFRS comprehensively recorded the epidemiology, management, and outcomes of OPF, providing a valuable resource for understanding the burden and therapeutic responses of OPF patients.

### 2.2 Study design and participants

**Figure 1:**
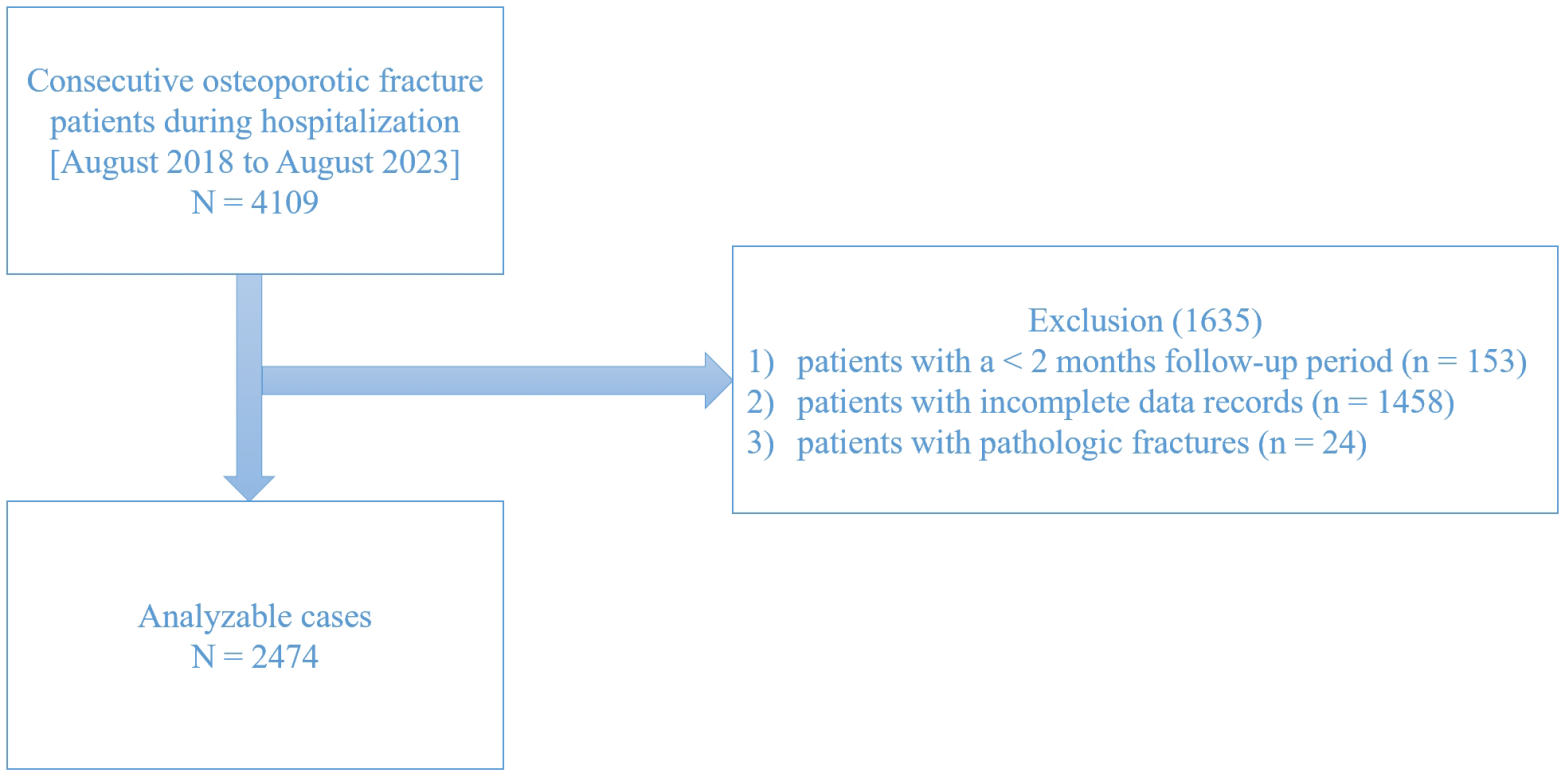
Study flow chart.

This retrospective analysis evaluated OPF patients aged ≥ 50 years who underwent surgical intervention between August 2018 and August 2023. To ensure data integrity and accuracy, patients (1) with a < 2 months follow-up period (n = 153), (2) with incomplete data records (n = 1458), and (3) with pathologic fractures (n = 24) were excluded from the study. In total, 2,474 patients were enrolled for investigation (**Figure 1**).

### 2.3 Patient’s record and data collection

The participants were recruited from the Kunshan Regional Health Registration Platform (RHRP). To collect the follow-up data, the AKHJU FF Registration System (FFRS) was linked with the RHRP using unique patient or hospital identifiers, along with diagnostic details and dates of surgery, admission, and discharge.

This retrospective observational study was authorized by the Affiliated Kunshan Hospital of Jiangsu University (approval number 2024-03-053-K01) and followed the Declaration of Helsinki. Because of its observational nature and anonymous data collection, the requirement of a signed consent form was waived.

### 2.4 Study exposure variable

NEU count at admission was set as the exposure variable. Furthermore, the baseline levels of NEUs were assessed *via* the Sysmex XN-10 automated hematology analyzer (Sysmex Corporation, Kobe, Japan) (16). To clarify ascertainment, admission NEU was obtained as part of the institutional preoperative laboratory protocol on the admission date and prior to surgery whenever feasible.

### 2.5 Study endpoint

The primary objective of this study was to identify the incidence of recurrent fractures, defined as fractures that require hospitalization and surgical intervention in the Kunshan area. A washout period between the initial OPF and any subsequent recurrence was set as 15 days. Early postoperative fractures are predominantly peri-operative periprosthetic or missed intraoperative injuries and clinical readmissions cluster within the first 1–2 weeks after hip/femur fracture, supporting the use of a short window to exclude index-episode events (17, 18). This research analyzed wrist, hip, proximal humerus, or vertebrae refracture cases. These recurrences were diagnosed based on the ICD-10 criteria using codes beginning with S52, S22, S42, S32, or S72. The data on recurrent fracture cases was sourced from the RHRP in Kunshan, China. The primary event of interest was refractures, and the follow-up duration was described as the time from the date of discharge after the first fracture to the date of the second fracture, relocation of the patient out of the area, or the end of the study period on August 30, 2023. It is important to note that while refractures were captured using administrative ICD-10 codes, all patients included in the study underwent radiographic evaluations as part of their clinical diagnosis and surgical treatment. The ICD-10 coding information was derived from our hospital’s inpatient surgical information system, ensuring thorough oversight and accuracy in capturing these events.

### 2.6 Covariates

The study participant’s baseline characteristics included creatinine (Cr), body mass index (BMI), age, Charlson Comorbidity Index (CCI) scores (0, 1 - 2, or ≥ 3), uric acid (UA), fracture category (femoral neck/thoracic vertebrae/proximal humerus//trochanteric/lumbar vertebrae/radius/subtrochanteric), gender, blood urea nitrogen (BUN), American Society of Anesthesiologists (ASA) classification (1 - 2/3 - 4), presence of diabetes mellitus (yes/no), smoking status (yes/no), hypertension (yes/no), and alcohol consumption (yes/no). To account for secondary prevention, we captured anti-osteoporotic therapies initiated around the index hospitalization, including calcium supplementation (e.g., Caltrate), bisphosphonates (alendronate, zoledronic acid), and teriparatide.

BMI was measured [ $BMI=\frac{weight\;(kg)}{height\;{\left(m\right)}^{2}}$] and blood was sampled at admission for assessing Cr, BUN, and UA using the Beckman AU5800 biochemical analyzer (Beckman Coulter, CA, USA) (19). Cr, BUN, and UA levels were measured using creatinine kinase (20), urease-glutamate dehydrogenase (21), and uricase-peroxidase methods (22), respectively.

For CCI scoring, specific weights were assigned to comorbidities according to their relative influence on patient mortality (23). The analyzed comorbidities included diabetes, renal disease, cancer, cardiovascular disease, *etc.*, and each was assigned a weight in 1–6 range. A higher weight indicated potentially higher effects on mortality. The patient’s total CCI score was measured by summing the individual weights and indicating the overall burden of comorbidities, thereby helping clinicians and researchers evaluate their impact on mortality or post-surgical complications. ASA classification is based on the patient’s preoperative physical health evaluation by anesthesiologists (24). Patients are categorized according to their underlying disease severity and the possible effect on anesthetic management. ASA classification and CCI scoring indicated the physical status and comorbid conditions of patients, respectively.

Smoking status was categorized as former or current based on the criteria if the individual had smoked within the past 12 months. Alcohol consumption was described as drinking at least once a week during the past 12 months.

### 2.7 Statistical analyses

#### 2.7.1 Patient profiles

Patient’s clinical, demographic, and laboratory data for continuous variables were indicated as medians with interquartile ranges (Q1-Q3) or means and standard deviations (SD). Whereas the categorical data was reported as frequencies and percentages. Pearson’s chi-squared test or Fisher’s exact test was employed for categorical variables to elucidate the variations in demographic characteristics between the validation and development cohorts. For normally distributed continuous variables, the independent samples t-test was carried out, while for non-normal distributions, the Mann-Whitney U test was performed.

#### 2.7.2 Model development

Cox proportional hazards regression models were employed to independently identify the link between the levels of NEUs and the risk of recurrent fractures over 5 years, adjusting for covariate’s effects. The results of Models 1, 2, and 3 were compared. First, the variance inflation factor (VIF) was employed for the collinearity diagnostics. Then, the necessity for covariate adjustment was elucidated based on the following criteria: In Criterion 1, covariates were introduced into the basic model (which initially comprised only NEU levels and fracture risk, without any covariates) or removed from the full model (which comprised all possible covariates such as ASA, gender, Cr, age, UA, fracture category, BMI, CCI, hypertension, BUN, smoking status, alcohol consumption, diabetes, calcium supplementation, bisphosphonates, teriparatide, and NEU levels along with fracture risk). The objective of this adjustment was to produce at least a 10% change in the odds ratio (OR). Criterion 2 included covariates that met Criterion 1 or indicated a *P*-value < 0.1 in univariate models (25). Furthermore, Model 1 was unadjusted, Model 2 was adjusted for age and BMI, while Model 3 included further adjustments based on Criterion 1 or 2, encompassing age, Cr, BUN, gender, UA, fracture type, BMI, ASA, hypertension, CCI, smoking status, diabetes, alcohol consumption, calcium supplementation, bisphosphonates, and teriparatide. Since the gender-specific characteristics were related to NEU levels and fracture risk, all analyses were stratified by gender to determine gender interactions. To maximize statistical power and minimize bias that might occur if patients with missing data were excluded from analyses, we used multiple imputation, based on 5 replications and a chained equation approach method in the R MI procedure, to account for missing data.

This investigation determined the link between NEU levels and the risk of recurrent fractures over a 5-year period using two distinct models: Model A (a linear model) and Model B (a piecewise nonlinear model). The selection of the superior model was made based on the *P-value*, where a *P < 0.05* indicated that the nonlinear model better represented the aforementioned association. In piecewise nonlinear models, restricted cubic spline functions are used for smooth curve fitting to measure thresholds. If the smoothing spline indicated a clear inflection point, recursive techniques were employed to automatically calculate the best-fit model *via* maximum likelihood estimation (26). To assess the stability of the derived turning points, we employed the Bootstrap method, conducting **≥** 1000 resamples of the dataset. This resampling technique allowed us to estimate the 95% confidence intervals for the threshold values. The smoothing spline fit was evaluated to identify the relationship between NEU levels and the 5-year risk of recurrent fractures, providing a nuanced understanding of how alterations in NEU levels impact fracture risk.

We used Kaplan Meier survival curves to visualize time event patterns stratified by NEU tertiles and gender. The subjects were divided into three groups based on their NEU level (low, medium, high) and stratified according to gender. Use Log rank test to compare the differences in survival curves between different groups.

To reduce potential distortion of admission NEU by peri-operative complications, we conducted the following sensitivity analyses. First, we excluded patients with evidence of peri-operative infection or fever, defined by any of the following recorded during the index hospitalization or within 7 days before admission: ICD-10 codes indicating procedure-related or device-related infections (e.g., T81.4, T84.5–T84.7), systemic infection/sepsis (A40–A41), pneumonia (J10–J18, J69), urinary tract infection (N39.0), osteomyelitis (M86), or skin/soft-tissue infection (L00–L08), and, if available, measured fever (≥38.0°C) or initiation of intravenous broad-spectrum antibiotics within −48 h to +48 h of surgery. Second, to address very-early events likely attributable to the index episode, we re-estimated Cox models with delayed entry at day 30 and day 60 after discharge (i.e., risk time commenced at day 30/60). All models preserved the same covariate adjustments as the primary analysis and were stratified by sex.

To evaluate the impact of BMD and fall history on the risk of recurrent fractures, we included these two variables in the Cox proportional hazards model in the sensitivity analysis. Considering that missing data may affect the reliability of the results, we will only include patients with complete records to ensure the accuracy of the analysis.

### 2.8 Software

All the statistical assessments were carried out *via* Empower Stats (X&Y Solutions, Inc., Boston, Massachusetts, USA) and R software version 3.6.3 (http://www.r-project.org). The *P*-value of ≤ 0.05 indicated the significance threshold.

## 3 Results

### 3.1 Baseline features of two genders

In total, 2,474 patients passed the inclusion criteria of the study. **Table 1** indicates the features of female and male participants stratified into tertiles on the basis of their NEU counts. There were 34.20% (n = 846) male and 65.80% (n = 1628) females. The stratification revealed significant variations in BUN and UA levels across the tertiles, and some differences were observed in gender characteristics.

**Table 1:** Study participant’s characteristics based on neutrophil tertiles in different sexes.

Characteristics	Male			*P*-value ^a^	*P*-value ^b^	Female			*P*-value ^a^	*P*-value ^b^
	T1 (3.10-4.40 ×10^9^/L)	T2 (5.50-6.70 ×10^9^/L)	T3 (8.10-11.02 ×10^9^/L)			T1 (3.10-4.40 ×10^9^/L)	T2 (5.50-6.70 ×10^9^/L)	T3 (8.10-11.02 ×10^9^/L)		
N	263	293	290			554	538	536		
Age, mean ± SD, y	68.58 ± 12.21	66.40 ± 11.50	67.06 ± 11.54	0.084	0.109	69.62 ± 10.86	69.37 ± 10.75	69.23 ± 10.47	0.827	0.808
BMI, mean ± SD, kg/m2	23.10 ± 3.33	23.22 ± 3.04	23.32 ± 3.12	0.733	0.533	23.26 ± 3.42	22.98 ± 3.14	23.40 ± 3.50	0.114	0.242
Cr, mean ± SD, μmol/L	63.99 ± 22.80	65.43 ± 48.24	64.21 ± 22.04	0.860	0.733	64.83 ± 27.02	67.20 ± 44.31	67.68 ± 32.78	0.361	0.220
BUN, mean ± SD, mmol/L	5.77 ± 1.73	5.66 ± 1.79	6.27 ± 2.33	<0.001	0.003	6.21 ± 6.09	5.88 ± 2.45	6.28 ± 3.09	0.255	0.028
UA, mean ± SD, μmol/L	269.64 ± 81.96	270.10 ± 80.73	311.24 ± 101.32	<0.001	<0.001	270.42 ± 88.31	275.40 ± 86.03	309.03 ± 98.97	<0.001	<0.001
Fracture category, N (%)				0.679	–				0.809	–
Thoracic vertebra	31 (11.79%)	33 (11.26%)	39 (13.45%)			100 (18.05%)	99 (18.40%)	94 (17.54%)		
Lumbar vertebra	79 (30.04%)	89 (30.38%)	78 (26.90%)			175 (31.59%)	175 (32.53%)	172 (32.09%)		
Wrist	15 (5.70%)	23 (7.85%)	26 (8.97%)			27 (4.87%)	32 (5.95%)	28 (5.22%)		
Proximal humerus	25 (9.51%)	37 (12.63%)	29 (10.00%)			70 (12.64%)	82 (15.24%)	73 (13.62%)		
Femoral trochanteric/subtrochanteric	113 (42.97%)	111 (37.88%)	118 (40.69%)			182 (32.85%)	150 (27.88%)	169 (31.53%)		
CCI score category, N (%)				0.959	0.955				0.766	–
0	230 (87.45%)	260 (88.74%)	258 (88.97%)			496 (89.53%)	482 (89.59%)	487 (90.86%)		
1–2	31 (11.79%)	30 (10.24%)	30 (10.34%)			52 (9.39%)	53 (9.85%)	44 (8.21%)		
≥ 3	2 (0.76%)	3 (1.02%)	2 (0.69%)			6 (1.08%)	3 (0.56%)	5 (0.93%)		
ASA category, N (%)				0.856	–				0.826	–
1–2	221 (84.03%)	241 (82.25%)	241 (83.10%)			419 (75.63%)	401 (74.54%)	408 (76.12%)		
3–4	42 (15.97%)	52 (17.75%)	49 (16.90%)			135 (24.37%)	137 (25.46%)	128 (23.88%)		
Hypertension, N (%)				0.760	–				0.885	–
No	229 (87.07%)	256 (87.37%)	258 (88.97%)			473 (85.38%)	456 (84.76%)	460 (85.82%)		
Yes	34 (12.93%)	37 (12.63%)	32 (11.03%)			81 (14.62%)	82 (15.24%)	76 (14.18%)		
Diabetes, N (%)				0.828	–				0.154	–
No	256 (97.34%)	285 (97.27%)	280 (96.55%)			531 (95.85%)	512 (95.17%)	522 (97.39%)		
Yes	7 (2.66%)	8 (2.73%)	10 (3.45%)			23 (4.15%)	26 (4.83%)	14 (2.61%)		
Smoking status, N (%)				0.830	–				0.588	0.613
No	219 (83.27%)	241 (82.25%)	244 (84.14%)			552 (99.64%)	536 (99.63%)	532 (99.25%)		
Yes	44 (16.73%)	52 (17.75%)	46 (15.86%)			2 (0.36%)	2 (0.37%)	4 (0.75%)		
Alcohol consumption, N (%)				0.958	–				0.362	0.437
No	235 (89.35%)	260 (88.74%)	257 (88.62%)			553 (99.82%)	538 (100.00%)	534 (99.63%)		
Yes	28 (10.65%)	33 (11.26%)	33 (11.38%)			1 (0.18%)	0 (0.00%)	2 (0.37%)		
Calcium supplementation, N (%)				0.768	–				0.934	–
	218 (82.89%)	247 (84.30%)	238 (82.07%)			453 (81.77%)	438 (81.41%)	441 (82.28%)		
	45 (17.11%)	46 (15.70%)	52 (17.93%)			101 (18.23%)	100 (18.59%)	95 (17.72%)		
Bisphosphonates, N (%)				0.238	–				0.480	–
	177 (67.30%)	215 (73.38%)	198 (68.28%)			301 (54.33%)	294 (54.65%)	309 (57.65%)		
	86 (32.70%)	78 (26.62%)	92 (31.72%)			253 (45.67%)	244 (45.35%)	227 (42.35%)		
Teriparatide, N (%)				0.330	0.311				0.079	–
	262 (99.62%)	293 (100.00%)	290 (100.00%)			546 (98.56%)	537 (99.81%)	530 (98.88%)		
	1 (0.38%)	0 (0.00%)	0 (0.00%)			8 (1.44%)	1 (0.19%)	6 (1.12%)		

T3, third tertile, T2, second tertile, T1, first tertile, CCI, Charlson comorbidity index, SD, standard deviation, Cr, creatinine, BMI, body mass index, UA, uric acid, ASA, American Society of Anesthesiologists, BUN, blood urea nitrogen.

a *p*-value: t-tests and chi-square tests for continuous and categorical variables, respectively.

b *p*-value: Kruskal Wallis rank test for continuous variables, Fisher exacts for categorical variables with expects < 10.

### 3.2 Adjusted and unadjusted Cox proportional hazard regression models

The associations between NEUs and the 5-year refracture rate among different genders were analyzed (**Table 2**). A total of 116 refracture events were recorded among 2,474 individuals at risk. The analysis models (Models 1, 2, and 3) revealed that there was no significant association between NEU and refracture rates among females across all models. However, reduced NEU levels were substantially associated with reduced refracture rates in males, specifically in Model 3 (95% CI = 0.72–0.97; HR = 0.84; *P*-value = 0.019). Overall, it was found that an increase in NEU counts was modestly but statistically significantly linked with a lower risk of refractures.

**Table 2:** Associations of neutrophil and 5-year refracture rate in different sexes.

Sex	No. of events/no. of patients at risk (%)	Model 1^a^ HR (95% CI) *P*-value	Model 2^b^ HR (95% CI) *P*-value	Model 3^c^ HR (95% CI) *P*-value
Female	82/1628 (5.04%)	0.96 (0.89, 1.04) 0.306	0.96 (0.89, 1.04) 0.345	0.96 (0.89, 1.04) 0.347
Male	34/846 (4.02%)	0.84 (0.73, 0.97) 0.017	0.85 (0.74, 0.98) 0.024	0.84 (0.72, 0.97) 0.019
Total	116/2474 (4.69%)	0.93 (0.87, 0.99) 0.029	0.93 (0.87, 1.00) 0.041	0.93 (0.87, 0.99) 0.036

a No adjustment.

b Adjusted for BMI and age.

c Adjusted for Cr, fracture category, UA, ASA, hypertension, CCI, BMI, BUN, diabetes, smoking status, age, alcohol consumption, calcium supplementation, bisphosphonates, and teriparatide.

HR, hazard ratio, CCI, Charlson comorbidity index, Cr, creatinine, BMI, body mass index, UA, uric acid, ASA, American Society of Anesthesiologists, BUN, blood urea nitrogen.

### 3.3 Smoothed curve fitting and threshold analyses

**Figure 2:**
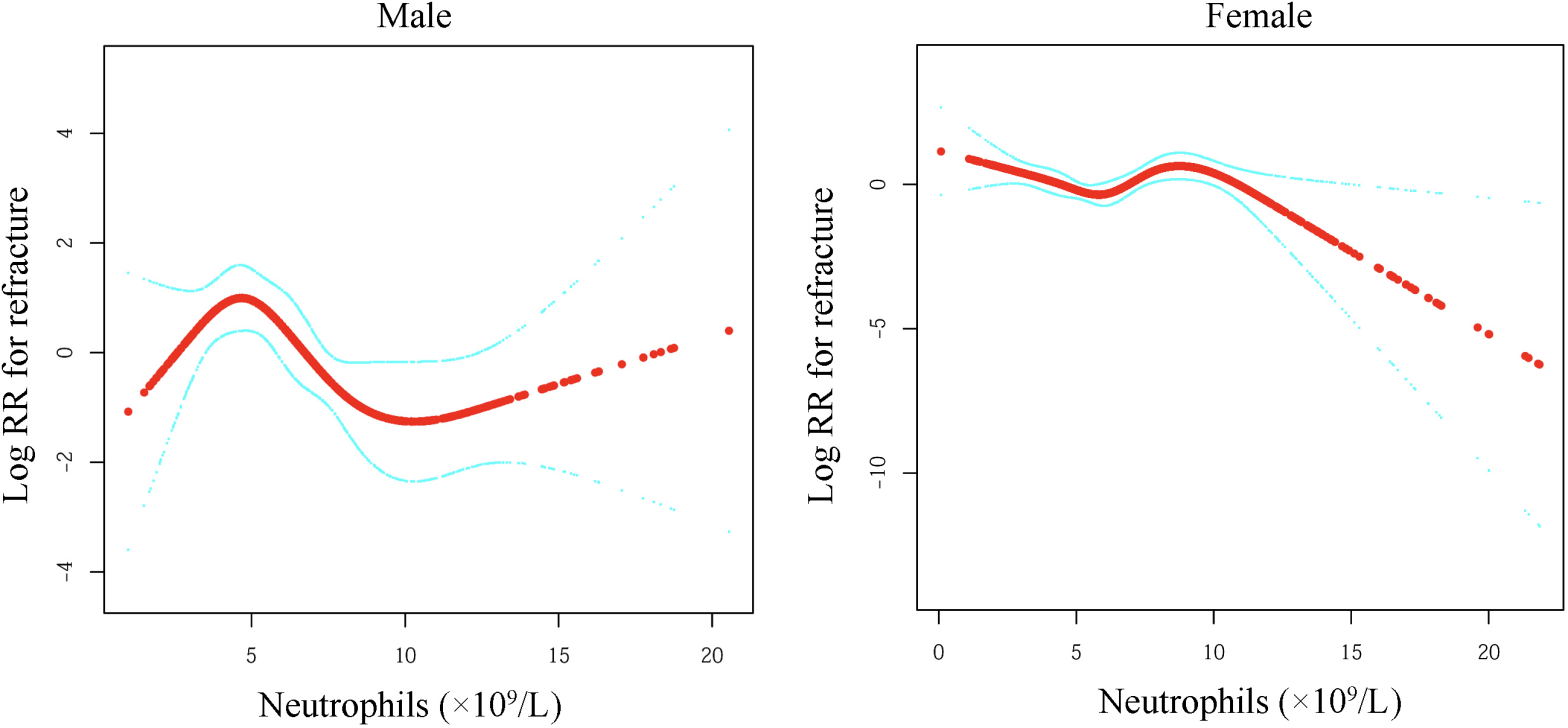
The relationship between neutrophil counts and fracture risk stratified by gender. Adjusted smoothed curves illustrating the association between neutrophil levels (×10^9^/L) and log relative risk (log RR) for fracture in males (left panel) and females (right panel). Generalized additive models revealed non-linear relationship in males. The thick red lines represent the primary association curves, while the light blue upper and lower curves denote 95% confidence intervals. Models were adjusted for Cr, fracture category, UA, ASA, hypertension, CCI, BMI, BUN, diabetes, smoking status, age, alcohol consumption, calcium supplementation, bisphosphonates, and teriparatide. CCI, Charlson comorbidity index; Cr, creatinine; BMI, body mass index; UA, uric acid; ASA, American Society of Anesthesiologists; BUN, blood urea nitrogen.

A nonlinear relationship was found between NEU levels and the 5-year refracture rate in males after adjusting for BMI, ASA, Cr, age, BUN, fracture category, UA, CCI, hypertension, smoking status, diabetes, alcohol consumption, calcium supplementation, bisphosphonates, and teriparatide (**Figure 2**). The threshold effect analysis was carried out within Model 3 to evaluate the association between NEUs and the 5-year refracture rate (**Table 3**), which showed a nonlinear relationship in male patients, as evidenced by a *P*-value < 0.05 in LRT. Furthermore, a three-piece nonlinear model was employed to determine the turning points (K) in the adjusted smooth curve of NEU at 4.4 (95% CI: 3.86–4.99) and 8.5 (95% CI: 7.32–9.21) ×10^9^/L for male patients. Moreover, a markedly negative correlation was identified between the 5-year refracture rate and NEU counts when the counts were within the range of 4.4 - 8.5 ×10^9^/L (95% CI = 0.37 - 0.86; HR = 0.57; *P*-value = 0.008). It was found that for an increase of 1 ×10^9^/L in NEU count, there is a 43% reduction in the risk of refracture in the adjusted model for male patients. These data revealed that the risk of refracture substantially decreases when NEU is within this range. However, there was no statistically significant difference when NEU counts fell below 4.4 (95% CI: 3.86–4.99) ×10^9^/L or above 8.5 (95% CI: 7.32–9.21) ×10^9^/L.

**Table 3:** Threshold effect analysis of the association between neutrophil and 5-year refracture rate using piece-wise linear regression.

Sex	Model 1^a^			*P*-interaction ^e^
	Male	Female	Total	
	HR (95% CI) *P*-value	HR (95% CI) *P*-value	HR (95% CI) *P*-value	
Model A^b^				0.105
One line effect	0.84 (0.72, 0.97) 0.019	0.96 (0.89, 1.04) 0.347	0.93 (0.87, 0.99) 0.036	
Model B^c^				0.157
Neutrophil turning point (K), per 1 ×10^9^/L	4.4 (3.86, 4.99), 8.5 (7.32, 9.21)	4.4 (3.86, 4.99), 8.5 (7.32, 9.21)	4.4 (3.86, 4.99), 8.5 (7.32, 9.21)	
< 4.4	2.09 (0.92, 4.76) 0.080	0.69 (0.48, 1.00) 0.050	0.94 (0.67, 1.31) 0.710	
4.4-8.5	**0.57 (0.37, 0.86) 0.008**	1.12 (0.90, 1.39) 0.317	0.92 (0.76, 1.12) 0.408	
> 8.5	1.10 (0.73, 1.64) 0.651	0.69 (0.48, 1.00) 0.052	0.80 (0.61, 1.05) 0.116	
LRT test^d^	0.035	0.039	0.678	

a Adjusted for age, BMI, Cr, BUN, UA, fracture category, CCI, ASA, hypertension, diabetes, smoking status, alcohol consumption, calcium supplementation, bisphosphonates, and teriparatide.

b Linear analysis, *P*-value < 0.05 showed a linear association.

c Three-piecewise linear analysis.

d *P*-value < 0.05 indicates that Model B is substantially different from Model A, suggesting a nonlinear association.

e *P*-value for the sex interaction, where *P <* 0.05 depicts a stronger interaction effect.

Bold values denote the primary findings of this study.

### 3.4 Impact of NEU tertiles and gender on refracture risk

**Supplementary Figure S1** shows the cumulative risk curve stratified by the tertiles of NEU and gender. In the NEU tertiles, the cumulative risk curves between the low NEU group and the moderate NEU group are relatively close, while the high NEU group shows a significantly higher cumulative risk, indicating that the risk of refracture increases significantly with increasing neutrophil levels. Specifically, the cumulative risk of the high NEU group was higher than the other two groups throughout the entire follow-up period, supporting the hypothesis of a positive correlation between NEU and the risk of recurrent fractures. In addition, gender based analysis showed that the cumulative risk curve of males was higher than that of females throughout the entire follow-up period, indicating that males have a significantly higher risk of refracture than females, highlighting the importance of gender in fracture risk management.

### 3.5 Sensitive analysis

To address potential misclassification of peri-operative complications, we implemented a 15-day washout period between index discharge and subsequent fractures, consistent with established hip-fracture epidemiological studies. Extending this washout to 30 and 60 days minimally altered event counts (**Supplementary Table S1**) and produced consistent effect estimates (**Supplementary Tables S1**, **S2**).

We performed two additional robustness checks to evaluate peri-operative influences on admission NEU. First, excluding patients with perioperative infections or fever (n=6) resulted in virtually unchanged estimates. Second, delayed entry analyses at 30 and 60 days post-discharge (left truncation) produced similar results, despite a reduction in person-time (**Supplementary Table S2**).

Across all sensitivity scenarios—including adjustment for anti-osteoporotic therapies and comorbidity burden (CCI)—the male-specific inverse association persisted. In linear models, HRs ranged from 0.83–0.86 (all *P* < 0.05), compared to 0.84 (95% CI: 0.72–0.97) in the main analysis. Women showed no significant association in any scenario (HRs: 0.96–0.97, all *P*>0.34) (**Supplementary Table S3A**).

Piecewise models confirmed the non-linear pattern in men, with consistent turning points at 4.4 and 8.5 ×10^9^/L. The protective association within the 4.4–8.5 ×10^9^/L range remained robust (HRs: 0.52–0.57, all *P* < 0.01). Women’s segment-specific estimates remained near null across all scenarios (**Supplementary Table S3B**).

We evaluated the relationship between NEU and 5-year re fracture rate using four models. Model 1 is the original adjusted model, and the results show that the HR for women is 0.96 (95% CI: 0.89, 1.04, *P* = 0.347), and for men it is 0.84 (95% CI: 0.72, 0.97, *P* = 0.019). In Model 2, we further adjusted for BMD and history of falls (HOF), and the results showed that the HR for females was 0.92 (95% CI: 0.76, 1.10, *P* = 0.352), while the HR for males significantly decreased to 0.73 (95% CI: 0.54, 0.99, *P* = 0.042). In Model 3, adjustments for bone metabolism markers P1NP and CTX were added, and the results showed that the HR for females was 0.93 (95% CI: 0.85, 1.02, *P* = 0.136), while the HR for males decreased to 0.80 (95% CI: 0.68, 0.95, *P* = 0.011). In Model 4, we further adjusted the anti-osteoporosis treatment and fall history status. The results showed that the hazard ratio (HR) for women was 0.96 (95% CI: 0.89, 1.04, *P* = 0.356), while the HR for men slightly decreased to 0.83 (95% CI: 0.72, 0.96, *P* = 0.014). Based on the results of various models, the HR of males is all below 1, indicating that NEU levels have a significant impact on the risk of recurrent fractures in males, while the HR of females does not change significantly and does not show statistical significance. These results validate the relationship between NEU and the risk of recurrent fractures, and emphasize the importance of gender in this relationship. (**Supplementary Table S4**).

The complete dataset generated through multiple imputation was compared with the observed complete case data, with relevant results presented in **Supplementary Table S5**. For females, the complete case analysis showed a hazard ratio (β) of 0.96 (95% CI: 0.89, 1.04, *P* = 0.347), while after applying multiple imputation, this hazard ratio increased to 0.98 (95% CI: 0.93, 1.04, *P* < 0.01). In the male cohort, the complete case analysis yielded a hazard ratio of 0.84 (95% CI: 0.72, 0.97, *P* = 0.019), which was adjusted to 0.87 (95% CI: 0.78, 0.97, *P* < 0.01) following multiple imputation. These results indicate that multiple imputation played a crucial role in reducing potential bias introduced by missing data, thereby enhancing the accuracy and reliability of the association between NEU levels and the risk of re-fracture across different genders.

These findings demonstrate that the observed male-specific inverse association between admission NEU and refracture risk is robust to various analytical specifications and unlikely attributable to peri-operative confounding or reverse causation.

## 4 Discussion

This preliminary population-based survey was designed to elucidate the association between NEU levels and the refracture risk in OPF patients. The data revealed a non-linear relationship in the adjusted model between NEU levels and refracture risk among male patients (95% CI = 0.72–0.97; HR = 0.84; *P*-value = 0.019). Furthermore, a sigmoidal (non-linear) relationship was found between the refracture risk and NEU levels, with adjusted smoothing curves identifying inflection points at 4.4 (95% CI: 3.86–4.99) ×10^9^/L and 8.5 (95% CI: 7.32–9.21) ×10^9^/L in the male cohort (95% CI = 0.37 - 0.86; HR = 0.57; *P*-value = 0.008). In addition, for an increase of 1 ×10^9^/L in NEU count, the risk of refracture was reduced by 43% in the adjusted model for male patients. However, no substantial associations were found between NEU levels and refracture rates in the female cohort across all models.

Compared to other studied populations, such as the elderly (27–29), this research specifically targeted a high-risk group comprising OPF patients over 50 years of age, requiring surgical intervention. The acquired results indicated the correlation between NEU levels and the incidence of refractures, specifically among male patients. Previous studies have largely overlooked gender differences; however, the present study highlights the significant gender-specific disparities. Here, a nonlinear relationship was identified between refracture rates and NEU levels in male participants, a discovery absent from prior research. Furthermore, the identified inflection points in NEU levels (4.4 (95% CI: 3.86–4.99) ×10^9^/L and 8.5 (95% CI: 7.32–9.21) ×10^9^/L) revealed that maintaining NEU counts within this range may protect against refractures in male OPF patients.

Previous literature was mainly focused on identifying the association of NLR with fracture risk (12, 30–33) and neglected the continuous variation in NEU levels. Although NLR can indicate alterations in inflammation levels (34), it is influenced by two independent metrics, which limits its effectiveness in assessing the direct relationship between NEU levels and fracture risk. Moreover, the previous literature did not adequately consider the protective role of NEUs in fracture healing and reducing the incidence of refractures (35).

This research study observed that patients with NEU levels outside the range of 4.4 - 8.5 ×10^9^/L had a higher risk of subsequent fractures. Therefore, the dual thresholds of 4.4 ×10^9^/L and 8.5 ×10^9^/L were recommended as critical indicators for enhanced vigilance in refracture risk analysis. These findings stimulate discussion on the ideal range of NEU levels for fracture prevention. Although the conventional normal range of NEUs is widely accepted (36), its actual impact on fracture risk remains elusive. In this investigation, the NEU range defined by the two inflection points differs from its conventionally defined normal values. Here, the data suggested that in male OPF patients, this specific range should be employed as a significant protective factor against fractures rather than relying solely on the traditional normal ranges.

However, the exact mechanisms responsible for the complex association between the refracture risk and NEU levels remain undetermined. Several studies have shown that NEU levels impact BMD. This relationship can be elucidated as follows: (1) NEUs influence bone density by upregulating RANKL expression, which is crucial for bone resorption and remodeling processes (37–39). RANKL is an important cytokine, and its increased expression induces osteoclast production, thereby directly participating in bone metabolism; (2) NEUs release various cytokines and chemical messengers during inflammatory responses (40), which may affect the health and metabolism of bone tissue; (3) NEU-derived catecholamines induce negative stress on bones (41). Furthermore, BMD is a crucial indicator for osteoporosis diagnosis and fracture risk assessment (42). In the elderly, bone density is a key determinant of fracture risk. Therefore, lower NEU levels may increase refracture risk *via* a mechanism involving reduced BMD.

NEU levels have also been linked with other risk factors for fractures. It has been observed that NEUs can directly or indirectly secrete substances that affect osteocytes and mesenchymal stem cells (43), thus mediating immune responses and promoting fracture healing. Moreover, increased local NEU counts after severe trauma have been found to impair bone repair (44). Furthermore, NEU depletion can adversely affect fracture healing outcomes (35). Therefore, maintaining an optimal number of NEUs is crucial for successful bone repair.

Our sex-stratified analyses revealed an inverse association between admission neutrophil counts (NEU) and refracture in men but not in women. Several factors may underlie this pattern. First, sex hormones differentially regulate neutrophil biology and bone remodeling. Estrogen signaling modulates neutrophil recruitment and effector functions and promotes osteoclast apoptosis via RANKL/OPG pathways, whereas androgens influence osteoblast/osteoclast activity through partly distinct mechanisms (45, 46). Such hormone-dependent “immuno-skeletal” coupling could yield sex-specific risk functions relating NEU to skeletal outcomes (47). Second, bone turnover and remodeling kinetics differ between men and postmenopausal women; faster turnover and distinct microarchitectural changes in women may attenuate the prognostic value of a single admission NEU measurement for subsequent refracture (48, 49). Third, innate immune responses exhibit sexual dimorphism—differences in neutrophil priming, trafficking, and NET formation have been reported—which could translate into distinct effects on callus biology and fracture healing (50, 51). Fourth, clinical care patterns may differ by sex (e.g., higher uptake of anti-osteoporotic therapy among women).

The findings of this study will help clinical and public health domains. It was observed that in male OPF patients when NEU levels are within the 4.4 - 8.5 ×10^9^/L range, the refracture risk is substantially reduced. The conventional normal range of NEUs does not indicate its actual impact on fracture risk. Here, it was validated that in male OPF patients, the above specific range may serve as a significant protective factor against fractures. Therefore, clinicians should not solely rely on the traditional normal range. Furthermore, fractures can alter NEU levels, which can be easily detected by simple blood tests. For every OPF patient requiring surgical treatment, a preoperative blood NEU test is essential. Measuring NEU levels can more accurately assess the risk of refracture in male OPF patients and enhance the effectiveness of preventive measures. For instance, implementing fracture liaison services can effectively manage and prevent fractures. Moreover, although biomarkers including MMP-9, CTK, CAII, p21, p53, and BAX affect fracture risk (52, 53), NEU-level testing is more convenient as surgeons typically do not perform specialized serological testing for these complex markers. In addition, the association between NEU levels and refracture rates is enduring. These findings demonstrate that NEU levels correlate with refracture rates over a follow-up period of 60 months. Thus, it was inferred that NEU levels are more suitable for predicting long-term refracture rates. It is therefore recommended that in clinical practice, NEU levels be used as a routine predictive marker for assessing long-term refracture risk.

## 5 Study strengths and limitations

This study has several advantages. Firstly, the sample size was large, and the follow-up period was long, which enhanced the result’s stability and reliability. Secondly, the included participants adequately represent the high-risk elderly Chinese population suffering from OPF, ensuring the study is representative. Thirdly, an open recruitment design was adopted in this research, which broadened the applicability and external validity of the acquired findings. This approach allowed the inclusion of a diverse population, thereby reducing selection bias. Since all eligible individuals could enroll, the underrepresentation of specific groups or the likelihood of systematic exclusion was minimized. Lastly, the prolonged follow-up period allowed for a more thorough assessment of refracture incidence.

However, this study has certain limitations. Firstly, the study could not establish a causal association between NEU levels and refracture risk, which warrants further research. Secondly, the study population was primarily acquired from Eastern China; therefore, the findings have geographical and ethnic limitations. Thirdly, only the NEU levels at the time of hospital admission were focused, and the dynamic changes in NEU levels were not considered. Therefore, these conclusions cannot be applied globally without further research. Fourthly, we were unable to specifically analyze the differences in hormone levels (such as testosterone and estradiol) related to gender. These biological factors may play an important role in revealing the role of gender in fracture risk mechanisms. Moreover, admission NEU may be transiently affected by acute stress responses or intercurrent infection. We sought to mitigate this by excluding peri-operative infection/fever and by initiating follow-up at post-discharge day 30/60 in sensitivity analyses. However, pre-admission inflammatory biomarkers (e.g., CRP, procalcitonin) and detailed antibiotic administration data were not available for all patients; thus, residual confounding from unmeasured infection activity cannot be ruled out. Comprehensive data on systemic corticosteroids and other immunomodulators were also lacking.

## 6 Conclusions

This retrospective cohort study indicated a pronounced sigmoidal correlation between NEU counts and the risk of refractures in male patients. Furthermore, it was found that maintaining NEU levels within the 4.4 - 8.5 ×10^9^/L range significantly reduces the risk of recurrent fractures, potentially serving as a protective factor in OPF men. This correlation was not observed in female patients. Therefore, NEU levels can serve as a biomarker for assessing fracture risk and complement traditional methods such as BMD measurements. Clinical application of NEU counts as biomarkers can improve the prognostication of fracture risks and refine management strategies for OPF patients. This approach would promote better patient management and assist in the effective allocation of healthcare resources, potentially reducing the associated healthcare costs.

The identification of a specific range of NEU counts correlated with reduced refracture risk and challenges the conventional range of ‘normal’ NEU levels in clinical practice. These findings provide the basis for research on the role of NEUs in bone health and disease, suggesting that a tailored therapeutic approach based on NEU levels could be beneficial in managing OPF patients.

In summary, this study provides information on the complex interactions between the immune system and bone health. Furthermore, it lays the groundwork for future research to explore the mechanisms underlying the protective effects of NEUs in bone repair and regeneration. Clinically, it furnished the evidence for routine NEU-level monitoring in high-risk OPF patients, advocating for a more nuanced approach to fracture prevention and management in the aging population.

## Data Availability

The original contributions presented in the study are included in the article/**Supplementary Material**. Further inquiries can be directed to the corresponding authors.

## Supplementary material

Supplementary Figure 1 Cumulative harm of neutrophil triad and gender to refractive fractures Left image: Cumulative risk curve of repeated fracturing stratified by the third quartile (low, medium, high) of neutrophils. These curves illustrate the relationship between neutrophil levels and the risk of recurrent fractures over time, with the red line representing the low quartile, the green line representing the middle quartile, and the blue line representing the high quartile Right figure: Cumulative hazard curve of repeated cracks stratified by gender. The red line represents the female group, while the green line represents the male group. These curves highlight the differences in the risk of recurrent fractures between genders during the observation period.
