# Supplementary figures and images for: Gender-specific associations between neutrophil levels and refracture risks: a retrospective cohort study
Source: Front Endocrinol (Lausanne). 2026 Jan 13;16:1625852. doi: 10.3389/fendo.2025.1625852 (PMC12834739; doi:10.3389/fendo.2025.1625852)

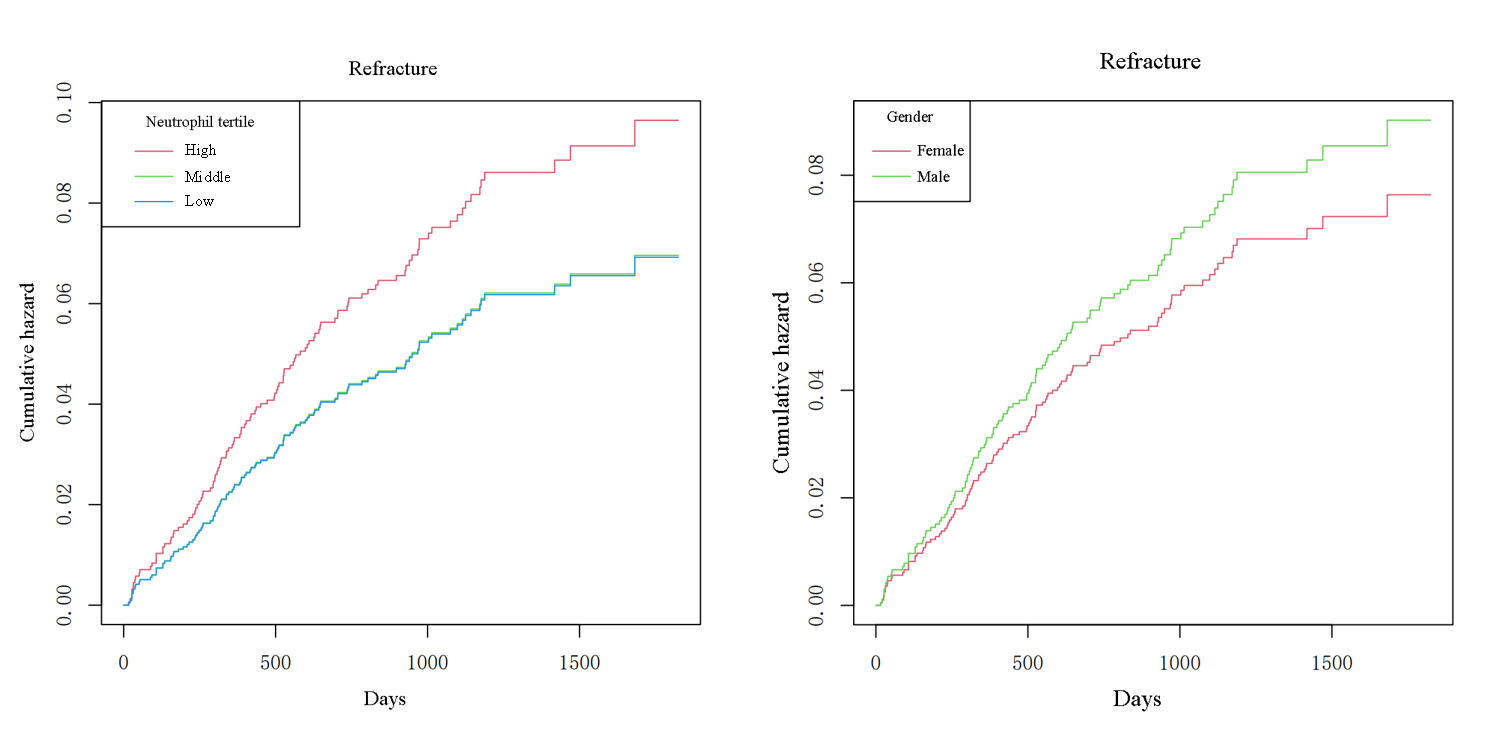

Supplement: Supplementary Figure 1 — Cumulative harm of neutrophil triad and gender to refractive fractures Left image: Cumulative risk curve of repeated fracturing stratified by the third quartile (low, medium, high) of neutrophils. These curves illustrate the relationship between neutrophil levels and the risk of recurrent fractures over time, with the red line representing the low quartile, the green line representing the middle quartile, and the blue line representing the high quartile Right figure: Cumulative hazard curve of repeated cracks stratified by gender. The red line represents the female group, while the green line represents the male group. These curves highlight the differences in the risk of recurrent fractures between genders during the observation period. [file Image1.jpg]
