# Supplementary material for: Gender-specific associations between neutrophil levels and refracture risks: a retrospective cohort study
Source: Front Endocrinol (Lausanne). 2026 Jan 13;16:1625852. doi: 10.3389/fendo.2025.1625852 (PMC12834739; doi:10.3389/fendo.2025.1625852)
Supplement: Supplementary file 3 [file Table1.docx]

**Table S1: Effect estimates by alternative washout window**s

| Washout window | No. of events/no. of patients at risk (%) | | Linear HR (95% CI) *P*-value ^a^ | | Piecewise HRs (95% CI) by segment *P*-value ^a^ | |
| --- | --- | --- | --- | --- | --- | --- |
|  | Female | Male | Female | Male | Female | Male |
| Primary (15-day) | 82/1628 (5.04%) | 34/846 (4.02%) | 0.96 (0.89, 1.04) 0.347 | 0.84 (0.73, 0.97) 0.019 | < 4.4: 0.69 (0.48, 1.00) 0.050; 4.4-8.5: 1.12 (0.90, 1.39) 0.317; > 8.5: 0.69 (0.48, 1.00) 0.052 | < 4.4: 2.09 (0.92, 4.76) 0.080; 4.4-8.5: **0.57 (0.37, 0.86) 0.008;** > 8.5: 1.10 (0.73, 1.64) 0.651 |
| 30-day | 80/1628 (4.91%) | 30/846 (3.55%) | 0.97 (0.90, 1.05) 0.472 | 0.82 (0.70, 0.95) 0.011 | < 4.4: 0.68 (0.47, 0.99) 0.044; 4.4-8.5: 1.16 (0.92, 1.44) 0.205; > 8.5: 0.69 (0.48, 1.00) 0.051 | < 4.4: 2.08 (0.97, 4.44) 0.058; 4.4-8.5: **0.53 (0.35, 0.79) 0.002;** > 8.5: 1.25 (0.97, 1.61) 0.086 |
| 60-day | 77/1628 (4.73%) | 27/846 (3.19%) | 0.97 (0.89, 1.05) 0.421 | 0.82 (0.70, 0.97) 0.021 | < 4.4: 0.69 (0.47, 1.00) 0.052; 4.4-8.5: 1.10 (0.88, 1.39) 0.400; > 8.5: 0.70 (0.48, 1.01) 0.054 | < 4.4: 1.90 (0.90, 3.99) 0.090; 4.4-8.5: **0.51 (0.33, 0.79) 0.003;** > 8.5: 1.25 (0.97, 1.61) 0.090 |

^a^ Adjusted for Cr, fracture category, UA, ASA, hypertension, CCI, BMI, BUN, diabetes, smoking status, age, alcohol consumption, calcium supplementation, bisphosphonates, and teriparatide.

Abbreviations: HR: hazard ratio, CCI: Charlson comorbidity index, Cr: creatinine, BMI: body mass index, UA: uric acid, ASA: American Society of Anesthesiologists, BUN: blood urea nitrogen.
