# Supplementary material for: Gender-specific associations between neutrophil levels and refracture risks: a retrospective cohort study
Source: Front Endocrinol (Lausanne). 2026 Jan 13;16:1625852. doi: 10.3389/fendo.2025.1625852 (PMC12834739; doi:10.3389/fendo.2025.1625852)
Supplement: Supplementary file 4 [file Table2.docx]

**Table S2. Sample size and events by analysis scenario**

| Population | Main analysis ^a^: N | Main analysis ^a^: Events | Excluding infection/fever ^b^: N | Excluding infection/fever ^b^: Events | Delayed entry Day 30 ^c^: N | Delayed entry Day 30 ^c^: Events | Delayed entry Day 60 ^d^: N | Delayed entry Day 60 ^d^: Events |
| --- | --- | --- | --- | --- | --- | --- | --- | --- |
| Overall | 116 | 2474 | 114 | 2468 | 109 | 2462 | 103 | 2456 |
| Male | 34 | 846 | 32 | 841 | 29 | 838 | 26 | 835 |
| Female | 82 | 1628 | 82 | 1626 | 80 | 1624 | 77 | 1621 |

^a^ Follow-up starts at discharge (day 0) with a 15-day washout; early events within 15 days are attributed to the index episode and not counted as refractures.

^b^ Individuals with peri-operative infection or fever recorded during the index hospitalization (and within 7 days pre-admission when available) were excluded, as defined by ICD-based algorithms (see Methods).

^c^ Follow-up commences at post-discharge day 30; only events occurring after the corresponding threshold contribute person-time and are counted.

^d^ Follow-up commences at post-discharge day 60; only events occurring after the corresponding threshold contribute person-time and are counted.

Abbreviations: N, number at risk.
