# Supplementary material for: Gender-specific associations between neutrophil levels and refracture risks: a retrospective cohort study
Source: Front Endocrinol (Lausanne). 2026 Jan 13;16:1625852. doi: 10.3389/fendo.2025.1625852 (PMC12834739; doi:10.3389/fendo.2025.1625852)
Supplement: Supplementary file 5 [file Table3.docx]

**Table S3 Cox proportional hazards estimate for NEU and refracture across robustness analyses**

A. Linear model ^e^

| Population | Scenario | HR (95% CI) | *P*-value |
| --- | --- | --- | --- |
| Male | Main analysis ^a^ | 0.84 (0.72–0.97) | 0.019 |
|  | Excluding peri-operative infection/fever ^b^ | 0.86 (0.74–0.99) | 0.040 |
|  | Delayed entry: Day 30 ^c^ | 0.83 (0.71–0.98) | 0.024 |
|  | Delayed entry: Day 60 ^d^ | 0.84 (0.72–0.99) | 0.043 |
| Female | Main analysis ^a^ | 0.96 (0.89–1.04) | 0.347 |
|  | Excluding peri-operative infection/fever ^b^ | 0.97 (0.89–1.04) | 0.375 |
|  | Delayed entry: Day 30 ^c^ | 0.97 (0.90–1.05) | 0.492 |
|  | Delayed entry: Day 60 ^d^ | 0.97 (0.89–1.05) | 0.440 |

B. Piecewise model ^e^

| Population | Scenario | Segment <4.4: HR (95% CI) | Segment 4.4–8.5: HR (95% CI) | Segment >8.5: HR (95% CI) |
| --- | --- | --- | --- | --- |
| Male | Main analysis ^a^ | 2.09 (0.92–4.76) | 0.57 (0.37–0.86) | 1.10 (0.73–1.64) |
|  | Excluding peri-operative infection/fever ^b^ | 2.14 (0.94–4.88) | 0.57 (0.37–0.88) | 1.10 (0.75–1.62) |
|  | Delayed entry: Day 30 ^c^ | 2.13 (1.00–4.54) | 0.53 (0.35–0.80) | 1.24 (0.96–1.60) |
|  | Delayed entry: Day 60 ^d^ | 1.96 (0.93–4.10) | 0.52 (0.33–0.80) | 1.23 (0.96–1.58) |
| Female | Main analysis ^a^ | 0.69 (0.48–1.00) | 1.12 (0.90–1.39) | 0.69 (0.48–1.00) |
|  | Excluding peri-operative infection/fever ^b^ | 0.70 (0.48–1.00) | 1.12 (0.90–1.40) | 0.70 (0.48–1.00) |
|  | Delayed entry: Day 30 ^c^ | 0.68 (0.47–0.99) | 1.16 (0.93–1.45) | 0.69 (0.48–1.00) |
|  | Delayed entry: Day 60 ^d^ | 0.69 (0.47–1.00) | 1.11 (0.88–1.39) | 0.70 (0.49–1.01) |

^a^ Follow-up starts at discharge (day 0) with a 15-day washout; early events within 15 days are attributed to the index episode and not counted as refractures.

^b^ Individuals with peri-operative infection or fever recorded during the index hospitalization (and within 7 days pre-admission when available) were excluded, as defined by ICD-based algorithms (see Methods).

^c^ Follow-up commences at post-discharge day 30; only events occurring after the corresponding threshold contribute person-time and are counted.

^d^ Follow-up commences at post-discharge day 60; only events occurring after the corresponding threshold contribute person-time and are counted.

^e^ Adjusted for Cr, fracture category, UA, ASA, hypertension, CCI, BMI, BUN, diabetes, smoking status, age, alcohol consumption, calcium supplementation, bisphosphonates, and teriparatide.

Abbreviations: NEU, neutrophil count, HR: hazard ratio, CI, confidence interval, CCI: Charlson comorbidity index, Cr: creatinine, BMI: body mass index, UA: uric acid, ASA: American Society of Anesthesiologists, BUN: blood urea nitrogen.
