# Supplementary material for: Gender-specific associations between neutrophil levels and refracture risks: a retrospective cohort study
Source: Front Endocrinol (Lausanne). 2026 Jan 13;16:1625852. doi: 10.3389/fendo.2025.1625852 (PMC12834739; doi:10.3389/fendo.2025.1625852)
Supplement: Supplementary file 6 [file Table4.docx]

**Table S4:** **Associations of neutrophil and 5-year refracture rate in different sexes.**

| Sex | Model 1^a^  HR (95% CI) *P*-value | Model 2^b^  HR (95% CI) *P*-value | Model 3^c^  HR (95% CI) *P*-value | Model 4^d^  HR (95% CI) *P*-value |
| --- | --- | --- | --- | --- |
| Female | 0.96 (0.89, 1.04) 0.347 | 0.92 (0.76, 1.10) 0.352 | 0.93 (0.85, 1.02) 0.136 | 0.96 (0.89, 1.04) 0.356 |
| Male | 0.84 (0.72, 0.97) 0.019 | 0.73 (0.54, 0.99) 0.042 | 0.80 (0.68, 0.95) 0.011 | 0.83 (0.72, 0.96) 0.014 |
| Total | 0.93 (0.87, 0.99) 0.036 | 0.86 (0.74, 1.00) 0.043 | 0.90 (0.83, 0.98) 0.013 | 0.93 (0.86, 0.99) 0.035 |

^a^ Adjusted for Cr, fracture category, UA, ASA, hypertension, CCI, BMI, BUN, diabetes, smoking status, age, alcohol consumption, calcium supplementation, bisphosphonates, and teriparatide.

^b^ Adjusted for Cr, fracture category, UA, ASA, hypertension, CCI, BMI, BUN, diabetes, smoking status, age, alcohol consumption, calcium supplementation, bisphosphonates, teriparatide, BMD, and HOF.

^c^ Adjusted for Cr, fracture category, UA, ASA, hypertension, CCI, BMI, BUN, diabetes, smoking status, age, alcohol consumption, calcium supplementation, bisphosphonates, teriparatide, P1NP, and CTX.

^d^ Adjusted for Cr, fracture category, UA, ASA, hypertension, CCI, BMI, BUN, diabetes, smoking status, age, alcohol consumption, calcium supplementation, bisphosphonates, teriparatide, anti-osteoporosis treatment status and HOF.

Abbreviations: HR: hazard ratio, CCI: Charlson comorbidity index, Cr: creatinine, BMI: body mass index, UA: uric acid, ASA: American Society of Anesthesiologists, BUN: blood urea nitrogen, BMD: bone mineral density, HOF: history of falls, P1NP: procollagen I N - terminal propeptide, CTX: carboxy-terminal cross-linked telopeptide of type 1 collagen.
