# Supplementary material for: Gender-specific associations between neutrophil levels and refracture risks: a retrospective cohort study
Source: Front Endocrinol (Lausanne). 2026 Jan 13;16:1625852. doi: 10.3389/fendo.2025.1625852 (PMC12834739; doi:10.3389/fendo.2025.1625852)
Supplement: Supplementary file 7 [file Table5.docx]

**Table S5** Relationship between neutrophil and 5-year refracture rate in different sexes with missing data comparing observed complete case data to results from pooling the datasets with imputed variables from multiple imputation

| SEX | Model 1^a^ | |
| --- | --- | --- |
|  | Complete case | Multiple imputation |
|  | N = 2474 | N = 4109 |
|  | β (95% CI) *P*-value | β (95% CI) *P*-value |
| Female | 0.96 (0.89, 1.04) 0.347 | 0.98 (0.93, 1.04) < 0.01 |
| Male | 0.84 (0.72, 0.97) 0.019 | 0.87 (0.78, 0.97) < 0.01 |
| Total | 0.93 (0.87, 0.99) 0.036 | 0.95 (0.91, 1.00) < 0.01 |

^a^ Adjusted for Cr, fracture category, UA, ASA, hypertension, CCI, BMI, BUN, diabetes, smoking status, age, alcohol consumption, calcium supplementation, bisphosphonates, and teriparatide.

Abbreviations: HR: hazard ratio, CCI: Charlson comorbidity index, Cr: creatinine, BMI: body mass index, UA: uric acid, ASA: American Society of Anesthesiologists, and BUN: blood urea nitrogen.
